# The Use of Complementary Health Approaches Among U.S. Veterans from 2002 to 2022: a Prevalence and Sex-Stratified Analysis

**DOI:** 10.1007/s11606-025-09620-5

**Published:** 2025-05-20

**Authors:** McKenzie Watson, Zhi Chen, Kaushal B. Nanavati, Jamie L. Romeiser

**Affiliations:** 1 Norton College of Medicine, SUNY Upstate Medical University, Syracuse, NY USA; 2 School of Public Health, University at Albany, SUNY, Albany, NY USA; 3 Department of Family Medicine, SUNY Upstate Medical University, Syracuse, NY USA; 4 Department of Public Health and Preventive Medicine, SUNY Upstate Medical University, Syracuse, NY USA

**Keywords:** veterans, complementary medicine, complementary health approaches, integrative medicine, whole health

## Background

U.S. veterans experience higher rates of chronic diseases.^1^ Evidence supporting the use of complementary health approaches (CHA) to help manage these conditions has grown in recent years, and utilization has risen among veterans.^2^ The Department of Veteran’s Affairs (VA) has expanded CHA services since 2016, a decision based on veteran demand.^2^

However, CHA use can occur within or outside of VA facilities.^2^ Therefore, trends in CHA use are not completely understood. To help address these gaps, we leveraged nationally representative survey data to estimate changes in the prevalence of using CHA among veterans from 2002 to 2022. Given past reported differences in CHA usage rates between males and females,^2,3^ we further stratified by sex to investigate these differences.

## Methods

We used data from the 2002 and 2022 National Health Interview Survey (NHIS), an annual, publicly available, nationally representative cross-sectional survey. All veterans surveyed with valid data for CHA use were included in the analysis. Veterans were defined as ever serving on active duty in the US Armed Forces, military reserves, or National Guard. All CHAs were assessed as self-reported use in the past 12 months. Prevalence estimates were generated for use of any CHA, any mind–body approaches (yoga, guided imagery, meditation), and any manipulative approaches (chiropractor, acupuncture, massage), and for all 6 individual approaches. Analyses were conducted using multilevel survey procedures in SAS 9.4 software. Estimates were further stratified by sex. Survey weighted prevalence estimates were then age and sex standardized where appropriate, using the 2010 US census population to allow for time comparisons.^4^ Time periods were compared using Cochran-Mantel–Haenszel tests, controlling for age, at the *p* = 0.05 significance level. Absolute change and relative change in prevalence were calculated.

## Results

**Figure 1:**
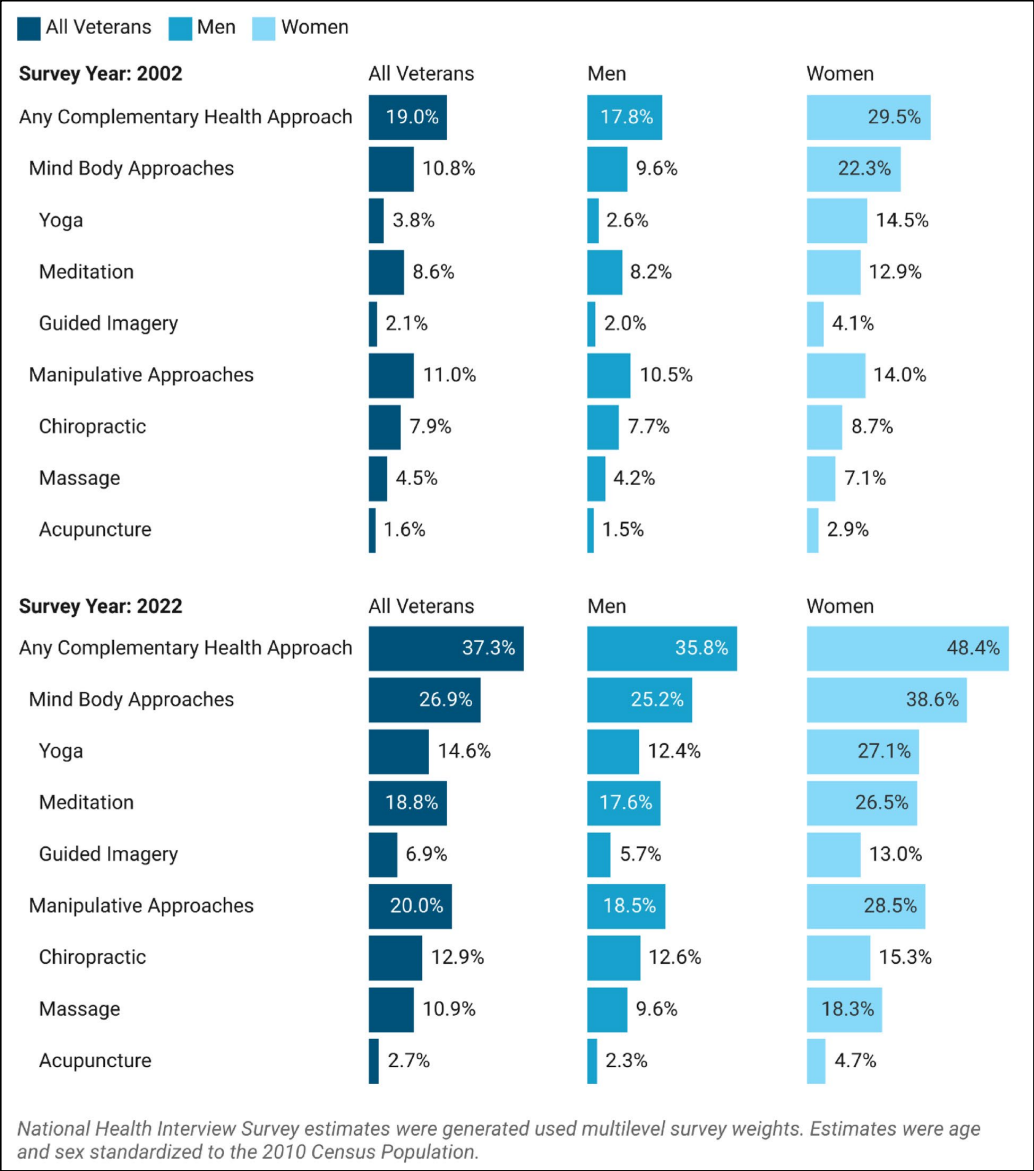
Complementary health approach use among veterans. Legend: Age and sex standardized prevalence estimates are presented for 2002 and 2022 for all veterans, and by sex. Cochran-Mantel–Haenszel tests (examining change by year while controlling for age) found significant differences in prevalence for all complementary health approaches (*p* < 0.05), with the exception of acupuncture in men and acupuncture in women.

**Figure 2:**
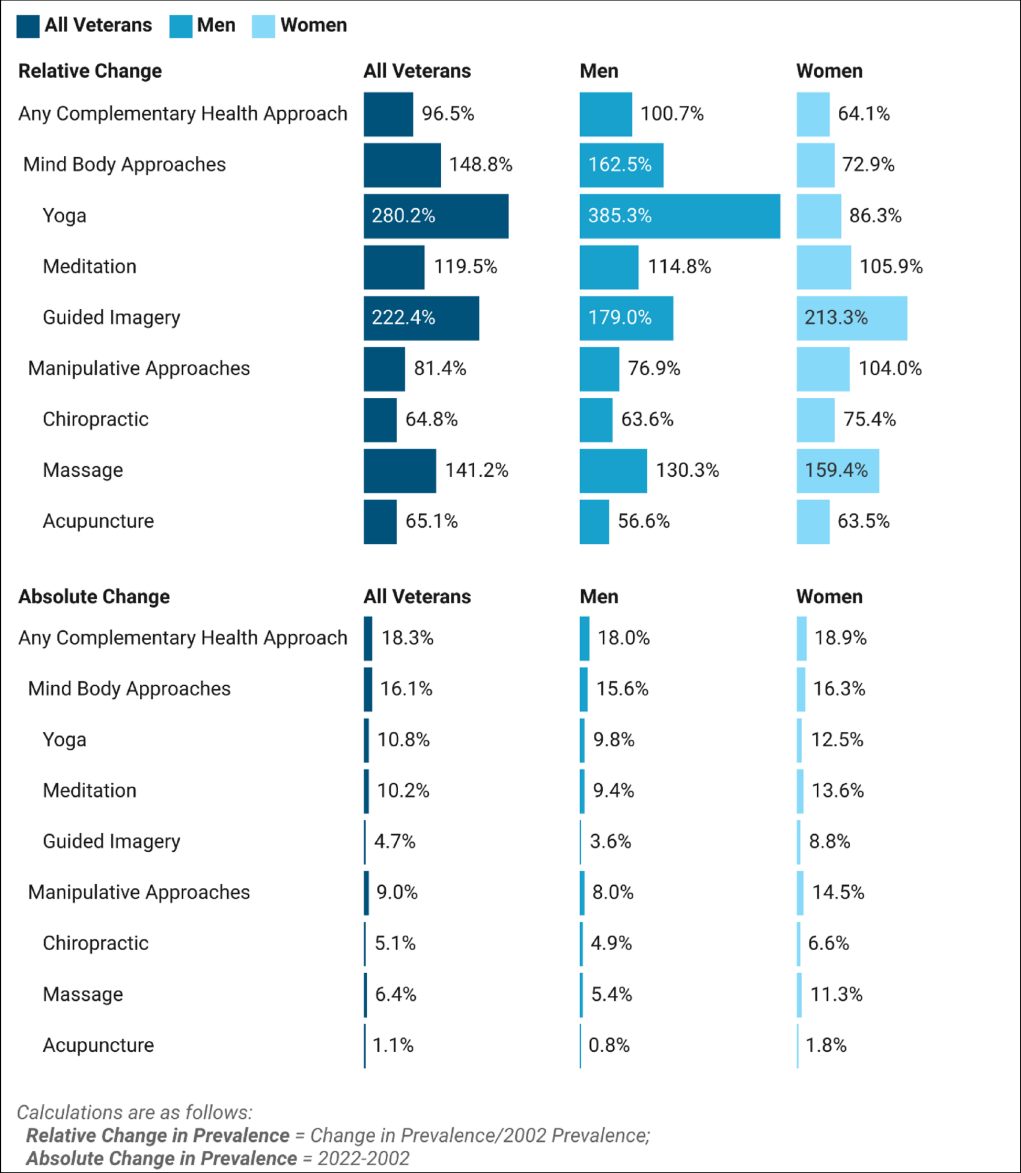
Change in Veteran use of complementary health approaches. Legend: Relative and absolute changes were calculated from 2002 to 2022 for all veterans, and by sex.

There were 3,428 (weighted *n* = 11,785,016) and 2,365 (weighted *n* = 9,217,300) veterans surveyed in 2002 and 2022, respectively. The prevalence of using any CHA increased from 19.0% (95%CI 19.0%−21.0%) to 37.3% (95%CI 34.0%−40.5%) over time (*p* < 0.001), (Fig. 1). Manipulative therapy use increased 9.0% (95%CI 6.0%−11.9%), while mind–body use rose 16.1% (95%CI 12.5%−19.7%). Prevalence of all 6 CHAs rose over time (*p* < 0.001), with the largest absolute changes seen for yoga (10.8%, [95%CI 8.0%−13.5%]) and meditation (10.2%, [95%CI 7.0%−13.4%]). This was consistent for both males and females. Females had higher baseline CHA use than males (29.5% [95%CI 25.8–33.2%] vs 17.9% [95%CI 15.6%−20.1%]), but absolute change was similar over time (+ 18.9%, [95%CI 10.7%−27.0%] vs + 18% [95%CI 13.7%−22.3%]), (Fig. 2). Overall relative change was greater in males compared to females (100.7% vs. 64.1%). In males, relative change was highest for yoga (385%) and guided imagery (179%), whereas in females, relative change was highest for guided imagery (213.3%) and massage (159.4%).

## Discussion

Overall prevalence of CHA use nearly doubled over the last 20 years among veterans, with females having higher baseline use but males experiencing higher relative increases. Mind–body approaches, particularly yoga for men, and guided imagery for women, saw the highest relative change. Use of meditation increased across both groups. Amongst the manipulative therapies, the greatest changes were seen in the use of massage therapy for both men and women.

One reason for this increase may be due to the incorporation of CHAs into pain management strategies.^4^ While male veterans experience a greater burden of chronic comorbidities,^5^ female veterans experience higher rates of pain-related and psychiatric diagnoses, which may explain their increased CHA use.^6^ Even though female veterans are more likely to be prescribed opioids than male veterans, they are more likely to seek out alternative pain-management strategies.^7^ Male veterans may be using CHAs more often due to VA promotion and expanded coverage.

Our study has limitations. Our data are cross-sectional, leading to potential recall bias. The NHIS does not survey those currently on active duty, which likely underestimates the true prevalence of use. Our results are limited to the 6 approaches that were collected. Finally, gender identity is not collected, which limits the analysis.

In summary, we found growth in reported use of CHAs over time in veterans, but growth differs for males and females, and by approach. These findings may help inform veteran outreach, engagement, referral, and care practices. Further analysis to assess effectiveness and motivations behind specific therapies may guide strategies for gender sensitive care, promotion, funding, and continued support for these approaches.

## Data Availability

All data are part of the National Health Interview Survey, which is collected by the National Center for Health Statistics in the Centers for Disease Control. Data are nationally representative, and made available to the public. More information, as well as the data, can be found here: https://www.cdc.gov/nchs/nhis/about/index.html.
